# Prevalence of Lameness in Dairy Cows and Associated Risk Factors at Hawassa Town Dairy Farms, Ethiopia

**DOI:** 10.1155/2024/2732333

**Published:** 2024-03-11

**Authors:** Abebe Tesfaye Gessese, Abayineh Ayele, Mebrie Zemene Kinde, Asefa Asmare

**Affiliations:** ^1^College of Veterinary Medicine and Animal Sciences, University of Gondar, P.O. Box 196, Gondar, Ethiopia; ^2^Faculty of Veterinary Medicine, Hawassa University, Hawassa, Ethiopia

## Abstract

Lameness is one of the greatest constraints on the productivity, health, and welfare of dairy cattle. A cross-sectional study was carried out from March 2021 to September 2021 in Hawassa town with the aim of assessing the prevalence and identifying the associated risk factors of lameness in dairy farms. The study was conducted on 440 animals belonging to 19 randomly selected intensive dairy farms. Data regarding lameness and its possible risk factors were collected both at animal and farm level using a questionnaire. The results showed that the overall prevalence of lameness was 10.2% (*n* = 45/440). The association of lameness prevalence with various risk factors including milking status, exercise, age, parity, milk yield, and lactation stage was statistically tested using logistic regression model. There was a significant variation in the prevalence of lameness (*P* < 0.05) between cattle with different milking status, age, parity, milk yield, and stage of lactation by the univariable analysis result. According to the multivariable analysis, only milk yield and lactation stage were statistically associated with the occurrence of lameness. Milking animals (8%) had higher prevalence of lameness than nonmilking (2.2%). The occurrence of lameness increased with milk yield. The highest prevalence of lameness was recorded in the early stage of lactation. Lameness was more frequent in hind limbs (6.6%) than in forelimbs (3.6%). The main causes of lameness observed in this study were both claw overgrowth 10 (2.3%), unequal claw size 10 (2.3%), solar ulcer 8 (1.8%), interdigital necrobacillosis 2 (0.5%), interdigital hyperplasia 2 (0.5%), and digital dermatitis 1 (0.2%). There was no means of early lameness diagnosis in 94.7% of farms. Lameness was found to be an important disease in dairy cows at Hawassa town. Prevention and early diagnosis leading to prompt treatment of lameness in cows should be part of dairy farm management practice.

## 1. Introduction

Dairy production plays a crucial role in Ethiopian livestock farming. The country possesses abundant and diverse livestock genetic resources, coupled with a variety of agroecologies suitable for dairy farming. The rising consumer demand for milk and its derivatives, favorable market conditions, and close proximity to international markets underscore the significant potential and opportunities for the development of the dairy industry in Ethiopia [1].

Despite this substantial potential, the dairy sector has not reached the anticipated level of development, and the overall productivity of dairy animals remains low [2, 3]. Various challenges, including issues like lameness [4–7], contribute to this situation. Consequently, there is a shortage in the supply of dairy products, necessitating the country to expend foreign currency on importing them from abroad [2].

Lameness, characterized by a departure from the typical walking pattern due to lesions, defects, injuries, diseases, or other factors affecting the limb or other parts of the body, is typically accompanied by pain or a certain level of discomfort [8]. Within the dairy industry, lameness stands out as a critical and pressing issue [9]. It is acknowledged as the foremost challenge impacting productivity, health, and welfare in dairy cattle.

Lameness represents a crucial production disease in dairy cows with notable economic implications [10]. In general, clinical incidences of lameness have a considerable negative impact on milk production, resulting in a reduction of 357 kg over a 305-day lactation period [11]. The onset of lameness is notably prevalent in the initial two months of a cow's first lactation, and the alarming statistic that 50% of animals experience chronic lameness during their life underscores its severity [12, 13]. Lameness emerges as a significant risk factor for culling throughout the lactation period. Cows treated for foot and leg problems at the onset and in the second month of lactation face a culling risk six to twelve times higher than that of healthy counterparts [14]. Beyond economic concerns, lameness in dairy cows raises serious welfare issues by causing pain and impeding the movement of the animals [15].

Numerous studies have indicated that lameness is correlated with various risk factors, including the type of flooring [16], seasonal variations [17], body condition [18], milk yield [19], parity [20], herd size [21, 22], breed [17, 23], age [24], and cleanliness of the floor and legs [17, 23].

Bovine lameness, ranking third globally in modern intensive dairy production following reproductive failure and mastitis, leads to reduced milk production, enhanced treatment expenses, increased culling rates, and prolonged calving intervals [25]. In Ethiopia, several studies highlight the significant impact of lameness in dairy cattle. An economic loss study conducted in Wolaita Sodo revealed that lameness resulted in a loss of 7.33 USD (125.30 ETB) per cow, attributed to decreased milk output and treatment costs as documented by Kifle [26]. Additionally, Sulayeman and Fromsa [4] determined a mean reduction of 1.63 litres in daily milk yield per cow in Hawassa dairy cattle. Among the lameness-positive animals, eight out of 15 were milking cows, while the remainder were nonmilking. The average daily milk production per cow decreased from 6.36 litres to 4.75 litres after the onset of lameness, indicating a mean loss of 1.63 litres in milk yield per cow per day.

Despite lameness posing significant economic losses and impacting the health and well-being of dairy cattle in Ethiopia, there is a scarcity of studies addressing the prevalence of lameness and its associated risk factors, particularly in Hawassa town. Furthermore, although lameness is influenced by various modifiable management practices, the last examination of lameness prevalence and associated risk factors for dairy cattle in Hawassa occurred in 2012 [4]. Therefore, the present study aims to update the prevalence and associated risk factors of lameness in dairy farms located in Hawassa town.

## 2. Materials and Methods

### 2.1. Study Area

The study was carried out in Hawassa town, which is located in Southern Ethiopia situated 270 km south of Addis Ababa. The area has a latitude of 7° 3′ 0″ N and a longitude 38° 28′ 0″ E on the escarpment of the Great Rift Valley. The altitude ranges from 1650 to 1700 meters above sea level. The average annual rainfall of the study area ranges from 800–1000 mm, and the mean temperature ranges from 11.14°C–29.1°C. The soil type of Hawassa town is lacustrine that is medium to fine textured and alluvial that includes clay, sand, and gravel. The area is mainly covered by dry savanna and bush type of vegetation including mainly short grasses and shrubs and to some extent eucalyptus, oak, and other indigenous and exotic plants [27]. The total livestock population of the study area constituted 1,721,341 cattle, 228,941 goats, 457,465 sheep, 57,643 horses, 54066 donkeys, 725, 5540 poultry, and 44,492 beehives [28].

### 2.2. Study Animals

The study was conducted on 440 Holstein Friesian dairy cattle belonging to 19 farms kept under intensive management system in Hawassa town. Each animal was identified by site of farm, age, parity, amount of milk per day, stage of lactation, and herd size using data files from the dairy personnel. The ages of the animals were determined primarily based on the information obtained from the animal owners and secondly by looking at the dentition pattern of the animals [29]. The floor system was concrete, and both roughage and concentrated feed were provided to the animals. All visited farms did not use bedding for their animals. All farms included in the study area have a practice of dung removal two or more times per day.

### 2.3. Study Design, Sampling Method, and Sample Size Determination

A cross-sectional study was carried out from March 2021 to September 2021 in Hawassa town. According to the information obtained from the agricultural office of Hawassa town, the town has 157 dairy farms. A list of all 157 farms was prepared, and 19 farms were selected using a simple random sampling, lottery technique. All animals of each selected farm were included in the study.

The sample size for the study was determined based on the description of Thrusfield [30] and considering as expected the prevalence of 50% as there was no previous study about prevalence of lameness in Hawassa town before this study, with the confidence interval of 95% and 5% required absolute precision. Then, the minimum required sample size was calculated using the following formula:

(1) $$\begin{array}{c} N=\frac{{\left(1.96\right)}^{2}\;P\exp\left(1-P\exp\right)}{d^{2}}, \end{array}$$

where *N* = sample size, *P* exp = expected prevalence, and *d* = required precision. By substituting the values in the formula and taking *d* = 0.05,

(2) $$\begin{array}{c} N=\frac{{\left(1.96\right)}^{2}0.5\left(1-0.5\right)}{{\left(0.05\right)}^{2}}=384. \end{array}$$

Even though the calculated sample size was 384, the study was conducted on a total of 440 animals by adding 15% to increase the precision.

### 2.4. Data Collection

A semistructured questionnaire which contained both animal and farm level questions was developed to collect data. Data regarding floor type, frequency of dung removal, and production status, animal's age, sex, lactation stage, type of feed, and site of lesion were collected. The questionnaire was developed based on previous studies [4, 31]. In order to assure the quality of the data, a pretest of data collection instrument was carried out on 5% of the total sample size outside the study area. Furthermore, all the animals in the selected farms were carefully observed and clinically examined for lameness. During the examination, the study animals were allowed to move and observed for any symptom of abnormal gait as described by Shearer et al. [32].

### 2.5. Data Analysis

The data collected in the paper format was transferred to and stored in Microsoft Excel database. Stata/MP software version 16 was used for the analysis of the data. The prevalence of lameness was presented using descriptive statistics. Logistic regression model was used to check the association of the abovementioned potential risk factors with the occurrence of lameness. Pearson's chi-square test was used to evaluate the association of different variables with the prevalence of lameness with treatment practice. In all statistical analysis executed, 95% confidence level and 5% precision were used and *P* value of less than 0.05 was considered as statistically significant.

## 3. Results

### 3.1. Sociodemography of Farm Workers

Male and female workers were involved to take care of the animals, and all workers have more than two years of working experience. The highest number of farm workers has an educational level of elementary school 31.57% (*n* = 6/19), followed by diploma and above 26.3% (*n* = 5/19), illiterate and high school 15.78% (*n* = 3/19), and the least number of workers was able to read and write 10.5% (*n* = 2/19). Dairy farming is the primary source of income for 16 farms out of 19 included in this study (Table 1).

### 3.2. Overall and Farm Level Prevalence of Lameness

By considering the total number of animals (*n* = 440) involved in the study, the overall prevalence of lameness was 10.2% (45/440). From the 19 observed farms, lameness was found in 89.47% (17/19) farms (Table 2).

### 3.3. Prevalence of Lameness with Associated Risk Factors

Table 3 displays the outcomes of a univariable logistic regression examination regarding the occurrence of lameness in dairy cows, considering different risk factors. With the exception of the permission for dairy animals to engage in exercise (*P* > 0.25), all the factors explored in the research, milking status, age, parity, milk yield, and lactation stage were determined to be statistically significant (*P* < 0.25). All the independent variables that demonstrated significance in the initial univariable analysis underwent an assessment for colinearity using Kruskal gamma statistics. Variables with gamma values falling between −0.6 and +0.6 were deemed suitable for inclusion in the multivariable logistic regression model. Consequently, milk yield and lactation stage were selected for the multivariable analysis. Both variables incorporated into the multivariable model, namely, milk yield and lactation stage, exhibited significance (*P* < 0.05) (Table 4). The Hosmer–Lemeshow goodness-of-fit test indicated that the model adequately fits the dataset (*χ*^2^ = 0.732; *P*=0.866).

### 3.4. Lesions That Caused Lameness and Site of Lesions

Various lesions which can cause lameness and their site were identified. All 45 (100%) lameness recorded were due to problems on the foot of animals. The lesions that were found causing lameness were claw overgrowth 10 (22.2%), unequal size claw 10 (22.2%), sole ulceration 8 (17.8%), interdigital hyperplasia and interdigital necrobacillosis 2 (4.4%), and digital dermatitis 1 (2.2%) as indicated in Table 5.

### 3.5. Prevalence of Lameness and Limbs Affected

We also described the proportion of lameness associated with the type of limbs. Lameness was observed due to problems from both limbs. However, hind limbs were more prone to lameness than forelimbs (Table 6).

### 3.6. Practice of Early Lameness Detection and Treatment

Out of the 19 farms observed, only one farm had the practice to detect early signs of lameness. The incidence of lameness in dairy cows is notably higher (10.59%) in farms lacking means for early recognition compared to those with such capabilities, where lameness is recorded at 7.7% (Table 7). Most of the treatments were carried out by veterinarians (16/19). The prevalence of lameness in dairy farms where farmers are responsible for treatment (11.11%) surpasses that in farms where veterinarians handle treatment (10.03%) (Table 7). The treatment success in the study area was 100%. Ten cows were culled in the last two years due to lameness.

## 4. Discussion

The current investigation revealed a lameness prevalence of 10.2% in dairy farms located in Hawassa town. This study highlights a significant and widespread occurrence of lameness in the study area, emphasizing the need for appropriate preventive and therapeutic measures. Notably, this prevalence is higher than the findings reported by Lobago et al. [33] who documented a lameness rate of 7.7% in clinically examined dairy cattle under urban and peri-urban production systems in the Addis Ababa milk shed. Similarly, Kifle [26] reported a lower lameness prevalence of 4.0% in Wolaita Sodo. In contrast, our study's prevalence is lower than that reported by Abunna et al. [31] in Bishoftu, where lameness was recorded at 13.9%.

Moreover, the prevalence of lameness found in the present study is lower than that reported in other countries, such as 36.8% in England and Wales [34] and 28.5% in Canada [35]. The variations in lameness prevalence among our study and those conducted in different countries could be attributed to differences in the management system, climate, study duration, cow productivity, and the methods employed for lameness detection and prevention. Geographical disparities and seasonal fluctuations in the incidence and prevalence of lameness are also evident, as indicated by Espejo et al. [36].

In the current study, the lameness prevalence varied among the farms, ranging from 0% to 33.3%, and there was a statistically significant correlation between the prevalence of lameness and the examined farms (*p* < 0.05). The difference in the prevalence of lameness between the farms might be due to the differences in management system and awareness of the negative impact of lameness.

This study examined the risk factors associated with lameness, considering both milking and nonmilking cows. The prevalence of lameness in milking cows was 35 (8%), while, in nonmilking cows without pregnancy, it was 10 (2.2%). The higher prevalence in milking cows is likely linked to the mobilization of fat from various tissues to support milk production, as suggested by Green et al. [37]. Consequently, the hypothesis was formulated that elevated milk yield may result in thinner digital cushions, exposing cows to conditions such as sole ulcers and white line disease [38]. The present study strengthened this hypothesis by evidencing that milk yield is significantly associated with the occurrence of lameness.

A higher prevalence of lameness, specifically 10.2%, was recorded in animals aged greater than two years compared to animals aged less than two years, where the prevalence was 0%. The findings of our study align with those of Manske et al. [39] who observed an increase in lameness with advancing age.

The occurrence of lameness in this study was higher in animals that yield more than 16 litters of milk per day (44.4%) compared to those produce less than 16 litters, and these might also be due to the loss of minerals such as calcium that have a great role in the strength of bone in animals.

In this study, the occurrence of lameness and the limbs affected was significantly associated, indicating that lameness was most common in hind limbs than in forelimbs possibly due to the fact that the hind legs were often contaminated with manure and kept wet. Hedges [40] also reported that on average, approximately 80% of lame cows are lame in the hind limbs. Singh et al. [41] also reached to similar findings from Punjab where the distribution of lameness in cattle was 28.9% in forefoot, 54.7 in hind feet, and 16.3% in both fore and hind feet. The same authors have also recorded more frequent foot abnormalities in the hind (80%) than in the fore (20%) feet in buffaloes.

Sadiq et al. [42] suggested that there was no significant link between the prevalence of lameness and parity. In contrast, our study demonstrated a noteworthy connection between lameness occurrence and parity. The higher prevalence in animals with more than two parities is believed to be attributed to their prolonged exposure to uncomfortable barn conditions and the absence of early lameness detection methods on the farm.

Van Amstel and Shearer [43] asserted that confinement on hard surfaces alone is sufficient to induce a mechanical form of laminitis, leading to subsequent claw overloading. Similarly, Barker et al. [34] found that housing dairy cows for 61 days or more were a significant risk factor associated with lameness prevalence in dairy herds in England and Wales. In contrast, Bicalho et al. [38] highlighted that lameness in dairy cows can manifest at any point during lactation, similar to many other diseases.

According to the present study, lesions that were found causing lameness were sole ulceration 8 (1.8%), digital dermatitis 1 (0.2%), claw overgrowth 10 (2.3%), unequal size claw 10 (2.3%), and interdigital hyperplasia and interdigital necrobacillosis 2 (0.5%). Highly prevalent lesions causing lameness in this study were unequal claw size and claw growth, and this might be due to lack of exercise and poor practice of hoof trimming.

## 5. Conclusion and Recommendations

The present study indicated a high and wide distribution of lameness that varied among the farms. High occurrence of lameness was recorded in cows with milking status, early lactation period, increased parity, and high milk yield. The present study showed that hind limbs of dairy cattle are more prone to foot lesions than the forelimbs. Poor means of recognizing early cases of lameness in the farms were another finding of the study. Therefore, providing awareness to farmers on the risk factors of lameness and management systems of dairy cattle is crucial to minimize lameness which is a serious welfare and economically important disease of dairy cows.

## Figures and Tables

**Table 1:** Gender and educational level of farm workers.

Respondents		Frequency	Percentage (%)
Gender	Male	11	57.89
	Female	8	42.11

Educational level	Illiterate	3	15.78
	Read and write	2	10.5
	Elementary	6	31.57
	High school	3	15.78
	Diploma and above	5	26.3

Income role	Primary source of income	16	84.2
	Not a primary source of income	3	15.78

**Table 2:** The prevalence of lameness in the individual examined farms.

Farm examined	Number of animals examined	Number of lame animals
Farm 1	10	2 (20.0%)
Farm 2	24	3 (12.5%)
Farm 3	10	2 (20.0%)
Farm 4	6	1 (16.7%)
Farm 5	6	1 (16.7%)
Farm 6	9	3 (33.3%)
Farm 7	52	4 (7.7%)
Farm 8	22	4 (18.2%)
Farm 9	15	4 (26.7%)
Farm 10	12	2 (16.7%)
Farm 11	24	4 (16.7%)
Farm 12	20	3 (15.0%)
Farm 13	20	2 (10.0%)
Farm 14	5	1 (20.0%)
Farm 15	6	0 (0.0%)
Farm 16	56	5 (8.9%)
Farm 17	129	2 (1.6%)
Farm 18	7	0 (0.0%)
Farm 19	7	2 (28.6%)
Total	440	45 (10.2%)

**Table 3:** Univariable logistic regression analysis of risk factors for the occurrence of lameness in Hawassa city dairy farms.

Risk factors	Level of factors	Number of animals visited	Number of positive animals (%)	Std. err.	COR	95% CI	*P* value
Milking status	Nonmilking	275	9 (3.3%)	—	—	—	—
	Milking	165	36 (21.8%)	0.388	0.121	0.057–0.259	<0.001

Exercise	Allow to exercise	249	22 (8.8%)	—	—	—	—
	Not allowed to exercise	191	23 (12%)	0.315	1.413	0.762–2.620	0.273

Age	<2 years	163	1 (0.6%)	—	—	—	—
	>2 years	277	44 (15.9%)	1.016	0.033	0.004–0.240	0.001

Parity	None	233	1 (0.4%)	—	—	—	—
	One	31	7 (22.6%)	1.019	61.755	8.380–455.114	<0.001
	≥2	176	37 (21%)	0.468	0.913	0.365–2.282	0.845

Milk yield	None	275	9 (3.3%)	—	—	—	—
	4 litre	43	5 (11.6%)	0.584	0.257	0.082–0.808	0.020
	4–8 litre	78	14 (17.9%)	0.449	0.155	0.64−0.373	<0.001
	8–16 litre	35	13 (37.1%)	0.487	0.057	0.022–0.149	<0.001
	>16 litre	9	4 (44.4%)	0.752	0.042	0.010–0.185	<0.001

Lactation stage	Nonlactating	275	9 (3.3%)	—	—	—	—
	<2 months	43	7 (16.3%)	0.534	0.174	0.061–0.496	0.001
	2–6 months	54	16 (29.6%)	0.451	0.80	0.033–0.195	<0.001
	6–9 months	35	6 (17.1%)	0.562	0.164	0.054–0.492	0.001
	>9 months	33	7 (21.2%)	0.544	0.126	0.043–0.365	<0.001

**Table 4:** Multivariable logistic regression analysis of potential risk factors for the occurrence of lameness in Hawassa city dairy farms.

Risk factors	Level of factors	Presence of lameness		AOR	95% CI	*P* value
		Yes	No			
Milk yield	None	9 (3.3%)	266 (96.7%)	—	—	—
	4 litre	5 (11.6%)	38 (88.4%)	0.230	0.060–0.888	0.033
	4–8 litre	14 (17.9%)	64 (82.1%)	0.147	0.045–0.480	0.001
	8–16 litre	13 (37.1%)	22 (62.9%)	0.051	0.015–0.175	<0.001
	>16 litre	4 (44.4%)	5 (55.6%)	0.038	0.006–0.230	<0.001

Lactation stage	Nonlactating	9 (3.3%)	266 (96.7%)	—	—	—
	<2 months	7 (16.3%)	36 (83.7%)	0.065	0.012–0.352	0.002
	2–6 months	16 (29.6%)	38 (70.4%)	0.028	0.006–0.135	<0.001
	6–9 months	6 (17.1%)	29 (82.9%)	0.056	0.011–0.290	0.001
	>9 months	7 (21.2%)	26 (78.8%)	0.038	0.006–0.230	<0.001

**Table 5:** Lesions that caused lameness in dairy cows in Hawassa town.

Lesion identified	Positive animals
Solar ulcer	8 (17.78%)
Digital dermatitis	1 (2.2%)
Interdigital necrobacillosis	2 (4.4%)
Interdigital hyperplasia	2 (4.4%)
Both claw overgrowth	10 (22.5%)
Unequal size claw	10 (22.5%)
Total	45 (100%)

**Table 6:** The prevalence of lameness and limbs affected in dairy cows in Hawassa town.

Limb affected	Number of lame animals
Right forelimb	6 (1.4%)
Left forelimb	4 (0.9%)
Right hind limb	10 (2.3%)
Left hind limb	14 (3.2%)
Both forelimbs	6 (1.4%)
Both hind limbs	5 (1.1%)
Total	45 (10.2%)

**Table 7:** Association of early lameness detection and treatment practice with the prevalence of lameness in dairy cows in Hawassa town.

Response		Presence of lameness			Percentage (%)	*X* ^2^ (*P* value)
		Yes	No	Total		
Means to recognize	Yes	4	48	52	7.7	0.42 (0.52)
	No	41	347	387	10.59	

Treatment condition	Farmer	10	80	90	11.11	0.09 (0.76)
	Veterinarian	35	314	349	10.03	

## Data Availability

The data supporting the current study are available from the corresponding author upon request.
